# A tool to assess fitness among adults in public health studies – Predictive validity of the FFB-Mot questionnaire

**DOI:** 10.1186/s12889-023-16174-w

**Published:** 2023-07-12

**Authors:** Alexander Woll, Laura Cleven, Darko Jekauc, Janina Krell-Roesch, Klaus Bös

**Affiliations:** Institute of Sports and Sports Science, Karlsruhe Institute of Technology, Engler-Bunte-Ring 15, 76131 Karlsruhe, Germany

**Keywords:** Motor fitness, Physical fitness, Health-related fitness, Exercise, Sports activity, Physical activity, Activities of daily living

## Abstract

**Background:**

Fitness has important implications for physical activity behavior and is associated with various health-related outcomes. It can be assessed through a test battery or a self-reported questionnaire. One example is the FFB-Mot (Funktionsfragebogen Motorik; engl. functional fitness questionnaire) which consist of 28 items to assess four components of fitness in adults: cardiorespiratory fitness/ endurance, muscular strength, gross motor coordination, and flexibility. The aims of this manuscript were to (1) provide an English-version of the FFB-Mot questionnaire (developed from the German-version using translation and back-translation) to the international community of researchers in the areas of physical activity, fitness and health in adults, and (2) examine the predictive validity of the FFB-Mot questionnaire in a large sample of community-dwelling adults.

**Methods:**

We used data from a longitudinal study in Germany with four measurement waves over a period of 18 years, with samples ranging between 310 and 437 participants (1572 adults in total, mean ages 46–58 years). To assess predictive validity, we calculated Pearson correlations between FFB-Mot data collected in 1997 and external health-related criteria (i.e., subjective health status, physician-rated health status, back pain, physical complaints and physical activity in minutes per week) collected in 2002, 2010, and 2015, and separately for males and females.

**Results:**

We observed correlations between higher FFB-Mot scores with better subjective health status (in 2002: males, r = 0.25; females, r = 0.18; in 2010: males, r = 0.29; females, r = 0.28; in 2015: males, r = 0.40), and higher physical activity (in 2002: males, r = 0.24; females, r = 0.25; in 2010: males, r = 0.30; females, r = 0.38; in 2015: females, r = 0.27). Higher FFB-Mot scores were also correlated with lower back pain (in 2002: males, r = -0.23; females, r = -0.25; in 2010: females, r = -0.22), less physical complaints (in 2002: males, r = -0.36; females, r = -0.24), and better physician-rated health status (in 2002: males, r = -0.41; females, r = -0.29, 2010: males, r = -0.38; females, r = -0.44; in 2015: males, r = -0.47).

**Conclusions:**

Our results suggest that the FFB-Mot to assess fitness in adults has predictive validity for health-related outcomes as indicated by significant correlations, albeit some effect sizes are small. The FFB-Mot may be used as one-time assessment of self-reported fitness, or for repeated testing to assess change of self-reported fitness over time and in different settings (e.g., public health research).

**Supplementary Information:**

The online version contains supplementary material available at 10.1186/s12889-023-16174-w.

## Background

Fitness is associated with both physical and mental health. For example, longitudinal research has shown that higher levels of fitness, particularly cardiorespiratory fitness (CRF), are associated with a decreased risk of stroke [1], cancer [2], cardiovascular disease [3], type 2 diabetes mellitus [4], sudden cardiac death [5], dementia [6, 7], body weight gain [8, 9], all-cause mortality [10] and certain mental health disorders [11]. Aside from CRF, other aspects of fitness are also critically important from a public health perspective. For example, decreased muscular strength is associated with higher fall risk in older adults [12].

Based on the theory of systematization of motor abilities by Bös and colleagues [13], CRF/ endurance, muscular strength, gross motor coordination, flexibility, and speed are considered main components of fitness. Briefly, the model distinguishes motor abilities into physical (mainly energetically-determined) and coordinative (mainly information-oriented) abilities. These are then further subdivided into five basic motor abilities, i.e., endurance (physical ability), muscular strength (physical ability), speed (physical and coordinative ability), gross motor coordination (coordinative ability), and flexibility (mainly anatomically determined) [13].

Thus, a comprehensive assessment of fitness in research studies in public health or related fields would require the use of a variety of fitness tests, or a carefully selected test battery including physiological (e.g., treadmill test to assess maximal oxygen uptake, VO_2_max), biomechanical (e.g., kinematic movement analyses), and motor performance measurements (e.g., balance test). However, this is often not feasible due to limited time, personnel or financial resources, particularly in large-scale, population-based observational studies [14, 15]. Hence, there is a need for affordable and easily available tools such as self-reported questionnaires.

To date, only few self-reported instruments to assess fitness are available. Examples include the Physical Activity Rating (PA-R; 16) or the International Fitness Scale (IFS; 17). Furthermore, a limited number of questionnaires have been designed to assess low-threshold fitness related to activities of daily living (ADL) such as the Barthel Index [18]. However, none of these tools comprehensively assesses various components of fitness such as CRF/ endurance, muscular strength, gross motor coordination, and flexibility. Furthermore, several questionnaires have only been validated for use in specific populations (e.g., older adults, persons with back pain), or lack published normative values.

In the past, our group has developed and published a simple, self-reported questionnaire entitled *Funktionsfragebogen Motorik* ((engl. functional fitness questionnaire; FFB-Mot) to assess four components of fitness (i.e., CRF/ endurance, muscular strength, gross motor coordination, flexibility) in German-speaking adults [19]. During the development phase of the questionnaire, several smaller studies were conducted. Briefly, questionnaire items were created and analyzed by a group of sports science experts, test-retest reliability was examined in a sample of 149 adults, and an expert panel study to rate acceptance and meaningfulness of the FFB-Mot items was carried out. The FFB-Mot shows a good test-retest reliability, with correlation coefficients ranging between r = 0.89–0.90, and a Cronbach’s alpha of 0.92. With regard to construct validity, an explorative factor analysis led to the identification of four factors (CRF/ endurance, muscular strength, gross motor coordination, and flexibility) with eigenvalues greater than 1.0. Furthermore, a multi-trait multi-methods (MTMM) analysis which compared FFB-Mot scales to the mean scores of an objective fitness test battery, revealed that the FFB-Mot questionnaire assesses the same constructs as the objective fitness test battery, with correlation coefficients ranging between r = 0.21–0.31 for CRF/ endurance, r = 0.38–0.50 for muscular strength, r = 0.53–0.58 for gross motor coordination, and r = 0.59–0.62 for flexibility [19]. The reader is referred to Additional File 1 for an overview of previous results on the quality of the FFB-Mot that were originally published in German. The FFB-Mot questionnaire may be used in various research settings, including in large-scale, population-based observational studies. It is feasible as a one-time assessment of self-reported fitness status (e.g., in cross-sectional/ case-control studies), or for repeated testing to assess change of self-reported fitness status over time (e.g., in longitudinal/ prospective cohort studies). Indeed, since the initial German publication in 2002, the FFB-Mot questionnaire has been used in various research studies in German speaking countries (e.g., 20–22).

In this manuscript, we aimed at providing an English-version of the questionnaire to the international research community that may be used in future studies in the areas of physical activity, fitness and health in adults. Furthermore, we aimed at expanding on our previous research by examining the predictive validity of the FFB-Mot (German version) in a large sample of community-dwelling adults over a time span of 18 years.

## Methods

### Fitness questionnaire (FFB-Mot)

The FFB-Mot questionnaire consists of a total of 28 items that describe certain everyday physical activities as indicators of four motor abilities as postulated by Bös and colleagues, such as carrying a heavy shopping basket upstairs across several floors (strength), or tying one’s shoes while standing (flexibility). Please refer to Table 1 for an overview of all items and corresponding motor abilities. Persons who complete the questionnaire respond to each item by rating on a 5-point Likert scale whether they would be able to manage carrying out the respective activity or not, with response categories ranging from “I am not able to carry out this activity” (1 point) to “I have no problem carrying out this activity” (5 points).

**Table 1:** The self-reported FFB-Mot questionnaire (28 items). This questionnaire asks you to rate how well you can manage carrying out the following 28 activities. Please respond to each item by selecting one of the five choices: I have no problem carrying out this activity; I have little problems carrying out this activity; I have moderate problems carrying out this activity; I have big problems carrying out this activity; I am not able to carry out this activity. Please answer all questions as best as you can. If you are unsure about which response to give for an item, please choose the one that appears most appropriate. Please answer according to what you think you are capable of rather than how often you have actually carried out the activity

#	Item (How well can you…)
**S-ADL**	sit on a chair and stand up without using your arms
**S1**	carry a heavy shopping basket upstairs across several floors (*)
**S2**	carry an object (e.g. a 12-pack beer) into the basement by going downstairs
**S3**	raise up your upper body from lying on your back to sitting without using your arms (sit-up) (*)
**S4**	lift a heavy suitcase above your head (e.g. putting it on the rack in a train)
**S5**	carry two heavy suitcases upstairs across several floors (*)
**S-Exercise**	lift a heavy object (e.g. dumb-bell) weighing more than your own body weight
**CRF-ADL**	take a brisk walk around several blocks
**CRF1**	climb multiple flights of stairs without resting
**CRF2**	walk briskly for 2 km (1.24 miles) without resting (*)
**CRF3**	run for 1 km (0.62 miles) without resting (*)
**CRF4**	run for 30 min without resting (approx. 5 km, (3.11 miles)) (*)
**CRF5**	run for 1 h without resting (approx. 10 km, (6.21 miles))
**CRF-Exercise**	run a marathon (42 km, (26,10 miles))
**F-ADL**	put on and take off a narrow sweater and socks by yourself
**F1**	reach the floor with your hands while sitting on a chair (*)
**F2**	tie your shoes while standing
**F3**	put one of your arms behind your back and slide your hand up toward your shoulder blade so that you can touch it
**F4**	bend down to touch the ground with your fingers while your legs are straight (*)
**F5**	touch your knees with your forehead while standing with your legs extended (*)
**F-Exercise**	bend backwards from standing position into a bridge position
**C-ADL**	walk down stairs without grasping the handrail
**C1**	do a one-leg stand without holding onto something (for at least 15 seconds) (*)
**C2**	do a somersault with your head touching the ground (*)
**C3**	dribble a ball while walking briskly
**C4**	jump over a fence of 1 m (3.28 feet) height, if necessary using your hands as support (*)
**C5**	ride a bike around a curve without using your hands
**C-Exercise**	do a cartwheel

Abbreviations: S1-5 = items to assess muscular strength; CRF1-5 = items to assess cardiorespiratory fitness/ endurance; F1-5 = items to assess flexibility; C1-5 = items to assess gross motor coordination; ADL = activities of daily living, items of additional ADL-scale; Exercise = items of additional Exercise-scale; (*) = items of short scale, Responses are rated on a scale ranging from “I am not able to carry out this activity” (1 point) to “I have no problem carrying out this activity” (5 points)

In accordance with the theory of motor abilities by Bös and colleagues, four different scales of the FFB-Mot have been developed and may be used depending on the aims of a given research study. The questionnaire with 28 items consists of 20 standard items with five items per dimension (i.e., CRF/ endurance, muscular strength, gross motor coordination, flexibility), plus four items for ADL with one item per dimension, and four items for persons who regularly engage in physical exercise also with one item per dimension.

#### Standard scale (20 items)

 Each of the four components of fitness (i.e., CRF/ endurance, muscular strength, gross motor coordination, flexibility) are assessed through five items. Items of the standard scale are shown in Table 1. Researchers may calculate a score for each fitness component separately, or an overall score for the entire 20 items scale. For the muscular strength subscale (S1-5), the possible score ranges from 5 to 25 points; for the CRF/ endurance subscale (CRF1-5), the possible score ranges from 5 to 25 points; for the flexibility subscale (F1-5), the possible score ranges from 5 to 25 points; and for the gross motor coordination subscale (C1-5), the possible score also ranges from 5 to 25 points. Thus, the possible total score for the 20 items standard scale ranges from 20 to 100 points. A higher score reflects a higher level of fitness.

#### Activities of daily living (ADL)-scale (four items)

In order to establish range for adults with limited fitness, an additional ADL-scale was created as follows: For each of the four components of fitness (i.e., CRF/ endurance, muscular strength, gross motor coordination, flexibility), one additional item was selected to reflect a low level of difficulty. Items of the ADL-scale are shown in Table 1. The possible score ranges from 4 to 20 points, with a higher score reflecting a higher level of fitness.

#### Exercise-scale (four items)

In order to establish range for adults with increased fitness, an additional Exercise-scale was created as follows: For each of the four components of fitness (i.e., CRF/ endurance, muscular strength, gross motor coordination, flexibility), one additional item was selected to reflect a high level of difficulty. Items of the Exercise-scale are shown in Table 1. The possible score ranges from 4 to 20 points, with a higher score reflecting a higher level of fitness.

#### Short-scale (12 items)

Three out of five items of the 20 items scale were selected for each of the four components of fitness (i.e., CRF/ endurance, muscular strength, gross motor coordination, flexibility) in order to create a 12 items short-scale. Items of the short-scale as shown in Table 1 are: S1, S3, S5, CRF2, CRF3, CRF4, F1, F4, F5, C1, C2, C4. The possible score ranges from 12 to 60 points, with a higher score reflecting a higher level of fitness.

#### English-version of the FFB-Mot

In a first step, the validated German version of the questionnaire was translated to English and back-translated to German by a licensed translator. In a second step, the translation and back-translation were verified by research scientists from Germany and the US in the areas of sports science and medicine. Please refer to Table 1 for the English version of the FFB-Mot.

### Study setting to assess predictive validity

The validation study reported in this manuscript was conducted using the German version of the FFB-Mot in the setting of the community-based longitudinal study “*Gesundheit zum Mitmachen”* in the town of Bad Schönborn in south-western Germany. The study had five measurement waves, i.e., 1992, 1997, 2002, 2010 and 2015. Participants were aged 33 to 56 years at baseline, and randomly selected from the resident register of Bad Schönborn in 1992. Furthermore, for every measurement wave, additional individuals aged 33 to 37 years also residing in Bad Schönborn but not yet participating in the study were invited for participation to prevent sample extinction. Participants who had already participated at an earlier measurement wave were re-invited to every measurement wave thereafter [9]. In total, data from 1572 participants were analyzed over the course of the study (i.e., 1997–2015). All participants provided written informed consent and participation was voluntary. The study was approved by the ethics committee of the Karlsruhe Institute of Technology, Germany, and was performed in accordance with the ethical standards as laid down in the 1964 Declaration of Helsinki. At each measurement wave, participants completed questionnaires about health status, physical activity, fitness and physical complaints that were derived from validated assessment tools. Furthermore, a physician evaluated the health status of each participant. Study assessments also include a motor performance assessment battery, assessment of body composition and blood work (data not reported in this manuscript). Data assessment took place from May to June for every measurement wave.

### Health-related criteria for the estimation of predictive validity

#### Subjective health status

Participants completed a self-reported health status questionnaire based on the works of Antonovsky [23, 24] and Becker [25, 26]. They were asked five questions, i.e., “How would you rate your health status?”; “How does your current health status impact your professional/ job performance?”; “How does your current health status impact your leisure time activities?”; “How would you rate your health status as compared to other persons of same age and sex?”; and “Has your health status declined over the past 5 years?” Responses were given on a five-point Likert scale ranging from 1 (e.g., very bad health status) to 5 (e.g., very good health status) [27]. For statistical analysis, an overall sum score (ranging from 5 to 25) was calculated to assess subjective health status, with a higher score indicating a better self-reported health status. The reliability of the scale is good with a Cronbach’s alpha of 0.82 [28].

#### Health status based on physician evaluation

In addition to the self-rated health status, the objective health status of each participant was assessed by a licensed physician. The physician rated the status of cardiovascular health, orthopedic health and neurological health on a 4-point scale (0, unimpaired; 1 light impairment; 2, moderate impairment; 3, severe impairment) based on medical history and physical examination. A sum score of all three domains (ranging from 0 to 9) was calculated to reflect objective health status, with a lower score indicating a better physician-rated health status.

#### Back pain

 To assess back pain, participants completed one question “Do you have back pain?” by responding on a five-point Likert scale of 0 (no pain) to 4 (severe pain).

#### Physical activity

Participants completed questions about their engagement in exercise-related physical activity (“Do you engage in any physical activity”? Yes or no), as well as questions about frequency (i.e., number of weekly exercise sessions) and duration (i.e., minutes per session) of their weekly physical activities carried out during leisure time and organized exercise/ sports [29]. For statistical analysis, a sum score of minutes of physical activities per week was calculated. The score has a high overall reliability, with a Cronbach’s alpha of 0.94 [28].

#### Physical complaints

The self-administered “Beschwerden-Liste-Revidierte Fassung” (B-LR) list of complaints (Zerssen Complaint List) was used to assess subjective impairments due to psychosomatic complaints (e.g., feeling of weakness, chest pain). It consists of 24 items, with response categories ranging from being free of any symptoms (score of 0) to having severe impairment (score of 3). For statistical analysis, a sum score was calculated ranging from 0 to 72, with a higher score indicating more severe physical complaints. The B-LR list has been used in several previous studies and is validated (e.g., split-half r = 0.93) [30, 31].

### Statistical analysis

Demographics of the study samples at all measurement waves (i.e., 1997, 2002, 2010, and 2015) were calculated using descriptive statistics, and presented as means and range for continuous variables, and frequency distribution (N and percentage) for categorical variables. To examine predictive validity, we calculated Pearson correlation coefficients between FFB-Mot data collected in 1997 (used as reference point = baseline measure; as the FFB-Mot questionnaire was first implemented in 1997) and external health-related criteria (i.e., subjective health status, physician-rated health status, back pain, physical complaints and physical activity minutes per week) collected in 2002, 2010, and 2015. Missing data were imputed with the sex specific mean of all valid data for each variable. All statistical analyses were conducted with IBM SPSS Statistics version 27, and a p-value of < 0.05 was used to determine statistical significance.

## Results

For an overview of demographics of the study sample at each measurement wave, please refer to Table 2.

**Table 2:** Demographics of study sample at each measurement wave

	Baseline 1997 (N = 437)	Follow-up 2002 (N = 429)	Follow-up 2010 (N = 310)	Follow-up 2015 (N = 396)
**Age in years, mean (range)**	45.73 (33–63)	49.23 (30–77)	57.51 (41–76)	54.33 (31–80)
**Male sex, N (%)**	218 (49.9)	229 (53.4)	156 (50.3)	176 (44.4)
**Health status, mean (range)**				
Subjective	17.14 (7–25)	16.96 (6–25)	17.11 (8–24)	17.62 (6–24)
Physician-rated	1.27 (0–6)	1.04 (0–8)	2.21 (0–8)	1.23 (0–8)
Back pain	1.51 (0–4)	1.53 (0–4)	1.5 (0–4)	1.96 (0–3)
Physical complaints	20.13 (0–51)	19.21 (0–61)	33.22 (0–69)	15.14 (0–54)
**Physically inactive, N (%)**	167 (38.2)	147 (34.3)	101 (32.6)	103 (26.0)
**Physical activity (min/week), mean (range)**	96.12 (0-1250)	105.11 (0-876.92)	137.68 (0-980)	122.64 (0-883.69)
**FFB-Mot, mean (range)**				
Standard scale	77.25 (22–100)	76.82 (20–99)	74.12 (30–100)	77.24 (24–100)
ADL-scale	19.13 (4–20)	18.83 (4–20)	18.97 (9–20)	19.17 (7–20)
Exercise-scale	7.5 (4–20)	7.78 (4–20)	7.29 (4–20)	7.73 (4–18)
Short scale	46.41 (13–60)	46.16 (12–60)	44.36 (18–60)	46.61 (14–60)

Abbreviations: ADL = activities of daily living; N = sample size; min = minutes; FFB-Mot = fitness assessed using self-reported FFB-Mot questionnaire. Subjective health status, possible range 5–25 points (higher score reflects better health); physician-rated health status, possible range 0–9 points (lower score reflects better health); back pain, possible range 0–4 points (lower score reflects less pain); physical complaints, possible range 0–72 points (lower score reflects less severe physical complaints), FFB-Mot standard scale, possible range 20–100 points (higher score reflects better fitness); FFB-Mot ADL-scale, possible range 4–20 points (higher score reflects better fitness); FFB-Mot exercise scale, possible range 4–20 points (higher score reflects better fitness); FFB-Mot short scale, possible range 12–60 points (higher score reflects better fitness)

The FFB-Mot standard scale at baseline (i.e., completed in 1997) was statistically significantly positively correlated with subjective health status (males, r = 0.25; females, r = 0.18), and physical activity (males, r = 0.24; females, r = 0.25) in 2002, indicating an association between higher self-reported fitness with higher subjective health status and physical activity. Similarly, the FFB-Mot standard scale at baseline (i.e., completed in 1997) was statistically significantly negatively correlated with back pain (males, r = -0.23; females, r = -0.25), physical complaints (males, r = -0.36; females, r = -0.24), and physician evaluation (males, r = -0.41; females, r = -0.29) in 2002, indicating an association between higher self-reported fitness with lower back pain, less physical complaints and better physician-rated health status.

When considering data from the follow-up assessment in 2010, there were statistically significant positive correlations between FFB-Mot completed in 1997 with subjective health status (males, r = 0.29; females, r = 0.28), and physical activity (males, r = 0.30; females, r = 0.38); as well as statistically significant negative correlations with physician evaluation (males, r = -0.38; females, r = -0.44), and back pain (females, r = -0.22).

Finally, when considering data from the follow-up assessment in 2015, there were statistically significant positive correlations between FFB-Mot completed in 1997 with subjective health status (males, r = 0.40), and physical activity (females, r = 0.27); as well as a statistically significant negative correlation with physician evaluation (males, r = -0.47). Please refer to Table 3 for an overview of all results related to predictive validity of the FFB-Mot.

**Table 3:** Predictive validity - Correlations between FFB-Mot (20-items standard scale) completed in 1997 and external health-related criteria in 2002, 2010 and 2015

Standard scale in 1997		Physical activity (min/week)		Subjective health status		Physician- rated health status		Physical complaints		Back pain	
		Male	Female	Male	Female	Male	Female	Male	Female	Male	Female
**2002**	r	**0.24** ^*^	**0.25** ^*^	**0.25** ^*^	**0.18** ^*^	**-0.41** ^*^	**-0.29** ^*^	**-0.36** ^*^	**-0.24** ^*^	**-0.23** ^*^	**-0.25** ^*^
	N	147	120	147	120	147	120	147	120	147	120
**2010**	r	**0.30** ^*^	**0.38** ^*^	**0.29** ^*^	**0.28** ^*^	**-0.38** ^*^	**-0.44** ^*^	-0.09	0.048	-0.04	**-0.22** ^*^
	N	113	103	113	103	113	103	113	103	113	103
**2015**	r	0.22	**0.27** ^*^	**0.40** ^*^	0.08	**-0.47** ^*^	-0.12	-0.09	-0.04	0.13	0.13
	N	67	57	67	57	67	57	67	57	67	57

Abbreviations: r = Pearson correlation coefficient, N = sample size, * = p < 0.05

## Discussion

Our current analyses show that the FFB-Mot questionnaire has predictive validity with regard to physical activity, subjective and physician-reported health status, physical complaints and back pain for both males and females, particularly when considering shorter (i.e., 5-years follow-up) rather than longer follow-up periods (i.e., 13- or 18-years follow-up).

These observations, together with our group’s previously reported findings [19], confirm that the FFB-Mot is a reliable and valid self-reported questionnaire to assess four components of fitness, i.e., CRF/ endurance, muscular strength, gross motor coordination, and flexibility in adults.

While objective tests to assess fitness remain the gold standard for research studies, the use of such batteries is often not feasible for a variety of reasons (e.g., limited time or financial resources, limited expertise to administer the tests). Since the FFB-Mot showed moderately significant associations with fitness batteries in prior research, the use of self-reported questionnaires such as the FFB-Mot may be a simple and cost-effective way of assessing fitness in research studies. To date, only few self-reported fitness questionnaires are available (please refer to Additional File 2). However, to the best of our knowledge, none of these tools comprehensively assesses various components of fitness such as CRF/ endurance, muscular strength, gross motor coordination, and flexibility. This is critically important as all components are associated with various physical or mental health-related outcomes, and thus a comprehensive assessment of fitness is highly relevant from a public health perspective. Furthermore, several questionnaires have only been validated for use in certain populations (e.g., older adults, persons with back pain), or lack published normative values. Therefore, the FFB-Mot expands on the existing body of fitness questionnaires, and can be considered an objective, reliable and valid tool to assess various components of fitness (i.e., CRF/ endurance, muscular strength, gross motor coordination, flexibility) in adults.

As the German version of the questionnaire has been available since 2002, several intervention (e.g., 32–34) and observational studies (e.g., 21, 35, 36, 20) conducted in German-speaking countries have used the FFB-Mot in recent years. In addition, the FFB-Mot was used as a reference tool for the validation of other scales, e.g., Assessment of Physical Activity-related Health Competence [22] and Physical Activity Biography [37].

The FFB-Mot questionnaire may be used in any research setting, including in large-scale, population-based observational studies in the areas of public health and other pertinent fields. Another strength is that it can be used as one-time assess-ment of self-reported fitness status (e.g., in cross-sectional/ case-control studies), or for repeated testing to assess change of self-reported fitness status over time (e.g., in longitudinal/ prospective cohort studies). Normative sex-specific values have been derived from a large, population-based sample of 12,306 persons (4,142 males) aged 16 to 99 years (mean age: 55 years), and are available to international investigators upon request from the corresponding author of this manuscript. In addition to research settings, the FFB-Mot may also be used in clinical practice, sports clubs or health centers, or community programs and outreach events to provide adult patients or the general public with a tool to assess and monitor fitness levels.

A main limitation is that the data on reliability and validity, including those presented in this manuscript, were derived using the German version of the FFB-Mot. Therefore, more studies, particularly in English-speaking countries, are needed to further assess the psychometric properties of the questionnaire. This will allow for a more comprehensive analysis and facilitate meaningful comparisons between the German and English language versions, and ultimately enhance FFB-Mot’s cross-cultural utility. Furthermore, future research endeavors should examine the feasibility of administering the FFB-Mot in ethnically and geographically diverse populations.

## Conclusions

In conclusion, the FFB-Mot is a simple and valid self-reported questionnaire to assess CRF/ endurance, muscular strength, gross motor coordination, and flexibility in the general adult population. Different scales are available and may be used depending on the aims of a given research study in public health or other related fields.

## Electronic supplementary material

Below is the link to the electronic supplementary material.

Supplementary Material 1

Supplementary Material 2

## Data Availability

The datasets generated and analyzed during the current study are not publicly available due to the strict ethical standards as required by the ethics committee of the Karlsruhe Institute of Technology, Germany; but are available from the corresponding author upon reasonable request from qualified researchers.

## Abbreviations

ADL: activities of daily living
CRF: cardiorespiratory fitness
FFB-Mot: Funktionsfragebogen Motorik (engl. functional fitness questionnaire)
