# Supplementary material for: A tool to assess fitness among adults in public health studies – Predictive validity of the FFB-Mot questionnaire
Source: BMC Public Health. 2023 Jul 12;23:1340. doi: 10.1186/s12889-023-16174-w (PMC10337075; doi:10.1186/s12889-023-16174-w)
Supplement: Supplementary file 1 — Supplementary Material 1 [file 12889_2023_16174_MOESM1_ESM.pdf]

ADDITIONAL FILE 2

Title: Self-reported motor fitness questionnaires

Description: Summary of existing self-reported motor fitness questionnaires (general motor fitness), low threshold fitness (for the assessment of activities of daily living), and physical activity.

Questionnaire	Reference
**Fitness**	
**FFB-Mot**	Bös, K., Abel, T., Woll, A., Niemann, S., Tittlbach, S., & Schott, N. (2002). Der Fragebogen zur Erfassung des motorischen Funktionsstatus (FFB-Mot). Diagnostica, 48(2), 101–111. https://doi.org/10.1026//0012-1924.48.2.101
**Non exercise fitness test** (PA-R – Physical Activity Rating)	Jackson, A. S., Blair, S. N., Mahar, M. T., Wier, L. T., Ross, R. M., & Stuteville, J. E. (1990). Prediction of functional aerobic capacity without exercise testing. Medicine and Science in Sports and Exercise, 22(6), 863–870. https://doi.org/10.1249/00005768-199012000-00021
**IFS** International Fitness Scale	Ortega, F. B., Ruiz, J. R., España-Romero, V., Vicente-Rodriguez, G., Martínez-Gómez, D., Manios, Y., Béghin, L., Molnar, D., Widhalm, K., Moreno, L. A., Sjöström, M., & Castillo, M. J. (2011). The International Fitness Scale (IFIS): Usefulness of self-reported fitness in youth. International Journal of Epidemiology, 40(3), 701–711. https://doi.org/10.1093/ije/dyr039
**Low-threshold fitness, mostly ADL**	
**Barthel Index**	Mahoney, F. I., & Barthel, D. W. (1965). Functional Evaluation: The Barthel Index. Maryland State Medical Journal, 14, 61–65.
**DASI** Duke Activity Status Index	Hlatky, M. A., Boineau, R. E., Higginbotham, M. B., Lee, K. L., Mark, D. B., Califf, R. M., Cobb, F. R., & Pryor, D. B. (1989). A brief self-administered questionnaire to determine functional capacity (the Duke Activity Status Index). The American Journal of Cardiology, 64(10), 651–654. https://doi.org/10.1016/0002-9149(89)90496-7
**FFbH-R** Hannover Functional Ability Questionnaire	Kohlmann, T., & Raspe, H. (1996). Der Funktionsfragebogen Hannover zur alltagsnahen Diagnostik der Funktionsbeeinträchtigung durch Rückenschmerzen (FFbH-R) [Hannover Functional Questionnaire in ambulatory diagnosis of functional disability caused by backache]. Die Rehabilitation, 35(1), I–VIII.

Questionnaire	Reference
**FIM** Functional Independence Measure, **FAM** Functional Assessment Measure	Keith, R. A., Granger, C. V., Hamilton, B. B., & Sherwin, F. S. (1987). The functional independence measure: A new tool for rehabilitation. Advances in Clinical Rehabilitation, 1, 6–18.<br><br>Hall, K. M., Hamilton, B. B., Gordon, W. A., & Zasler, N. D. (1993). Characteristics and comparisons of functional assessment indices. Journal of Head Trauma Rehabilitation, 8(2), 60–74. https://doi.org/10.1097/00001199-199308020-00008
**Physical activity**	
**Baecke Questionnaire**	Baecke, J. A., Burema, J., & Frijters, J. E. (1982). A short questionnaire for the measurement of habitual physical activity in epidemiological studies. The American Journal of Clinical Nutrition, 36(5), 936–942. https://doi.org/10.1093/ajcn/36.5.936
**BSA-F German „**Bewegungs- und Sportaktivitätsfragebogen“	Fuchs, R., Klaperski, S., Gerber, M., & Seelig, H. (2015). Messung der Bewegungs- und Sportaktivität mit dem BSA-Fragebogen. Zeitschrift Für Gesundheitspsychologie, 23(2), 60–76.
**CHAMPS** Physical Activity Questionnaire for Older Adults	Stewart, A. L., Mills, K. M., King, A. C., Haskell, W. L., Gillis, D., & Ritter, P. L. (2001). Champs physical activity questionnaire for older adults: Outcomes for interventions. Medicine and Science in Sports and Exercise, 33(7), 1126–1141. https://doi.org/10.1097/00005768-200107000-00010
**GPAQv2** WHO Global Physical Activity Questionnaire version 2	Armstrong, T., & Bull, F. (2006). Development of the World Health Organization Global Physical Activity Questionnaire (GPAQ). Journal of Public Health, 14(2), 66–70. https://doi.org/10.1007/s10389-006-0024-x
**Havard Alumni Questionnaire**	Paffenbarger, R. S., Wing, A. L., & Hyde, R. T. (1978). Physical activity as an index of heart attack risk in college alumni. American Journal of Epidemiology, 108(3), 161–175. https://doi.org/10.1093/oxfordjournals.aje.a112608
**IPAQ** International Physical Activity Questionnaire	Booth, M. (2000). Assessment of physical activity: An international perspective. Research Quarterly for Exercise and Sport, 71 Suppl 2, 114–120. https://doi.org/10.1080/02701367.2000.11082794
**MLTPAQ** Minnesota Leisure Time Physical Activity Questionnaire	Taylor, H. L., Jacobs, D. R., Schucker, B., Knudsen, J., Leon, A. S., & Debacker, G. (1978). A questionnaire for the assessment of leisure time physical activities. Journal of Chronic Diseases, 31(12), 741–755. https://doi.org/10.1016/0021-9681(78)90058-9
**Modified Baecke Questionnaire**	Voorrips, L. E., Ravelli, A. C., Dongelmans, P. C., Deurenberg, P., & van Staveren, W. A. (1991). A

Questionnaire	Reference
	physical activity questionnaire for the elderly. Medicine and Science in Sports and Exercise, 23(8), 974–979.
**PAR** seven days physical activity questionnaire	Sallis, J. F., Haskell, W. L., Wood, P. D., Fortmann, S. P., Rogers, T., Blair, S. N., & Paffenbarger, R. S. (1985). Physical activity assessment methodology in the Five-City Project. American Journal of Epidemiology, 121(1), 91–106. https://doi.org/10.1093/oxfordjournals.aje.a113987
**PASE** Physical Activity Scale for elderly	Washburn, R. A., Smith, K. W., Jette, A. M., & Janney, C. A. (1993). The Physical Activity Scale for the Elderly (PASE): Development and evaluation. Journal of Clinical Epidemiology, 46(2), 153–162. https://doi.org/10.1016/0895-4356(93)90053-4
**Zutphen Physical Activity Questionnaire**	Caspersen, C. J., Bloemberg, B. P., Saris, W. H., Merritt, R. K., & Kromhout, D. (1991). The prevalence of selected physical activities and their relation with coronary heart disease risk factors in elderly men: The Zutphen Study, 1985. American Journal of Epidemiology, 133(11), 1078–1092. https://doi.org/10.1093/oxfordjournals.aje.a115821
