# Supplementary material for: A tool to assess fitness among adults in public health studies – Predictive validity of the FFB-Mot questionnaire
Source: BMC Public Health. 2023 Jul 12;23:1340. doi: 10.1186/s12889-023-16174-w (PMC10337075; doi:10.1186/s12889-023-16174-w)
Supplement: Supplementary file 2 — Supplementary Material 2 [file 12889_2023_16174_MOESM2_ESM.docx]

**ADDITIONAL FILE 1**

**Title: Psychometric properties/ quality of the FFB-Mot questionnaire (as reported in Bös et. al, 2002 (19))**

**Objectivity:**

Sufficient objectivity due to high degree of standardization. Questionnaire was developed by a group of renowned sports scientists, and revised several times during expert panel studies.

**Reliability:**

*Test-Retest-Reliability*: (Assessed in 149 adults using repeated measurements with 2 weeks period in-between)

| FFB-Mot Scale | Females | Males |
| --- | --- | --- |
| Standard scale (20 items) | r = 0.89 | r = 0.90 |
| Muscular strength scale (5 items) | r = 0.93 | r = 0.90 |
| Cardiorespiratory fitness scale (5 items) | r = 0.83 | r = 0.89 |
| Flexibility scale (5 items) | r = 0.74 | r = 0.87 |
| Gross motor coordination scale (5 items) | r = 0.82 | r = 0.73 |
| Short scale (12 items) | r = 0.89 | r = 0.90 |
| ADL scale (4 items) | r = 0.78 | r = 0.82 |
| Exercise scale (4 items) | r = 0.77 | r = 0.82 |

ADL = activities of daily living; r = correlation coefficient

*Internal consistency:* (Assessed in 458 adults using Cronbach’s alpha)

Standard scale: α = 0.92

**Validity:**

*Validity of content:* Confirmed by expert rating.

*Validity of construction:* Classification of items to four dimensions was confirmed by factor analysis and expert rating. After using Multitrait-Multimethod-analysis which compared FFB-Mot scales to the mean scores of an objective fitness test battery, the requirements for convergent and discriminant validity are fulfilled.

|  |  | FFB-Mot | | | | | MTB | | | | |
| --- | --- | --- | --- | --- | --- | --- | --- | --- | --- | --- | --- |
|  |  | S | CRF | F | C | 28-Item | S | CRF | F | C | 28-Item |
|  | S | - | 0.50 | 0.55 | 0.53 | 0.76 | 0.38 | 0.03 | 0.43 | 0.38 | 0.43 |
|  | CRF | 0.63 | - | 0.43 | 0.48 | 0.79 | 0.29 | 0.21 | 0.33 | 0.34 | 0.46 |
| FFB-Mot | F | 0.64 | 0.53 | - | 0.53 | 0.76 | 0.39 | -0.40 | 0.62 | 0.31 | 0.51 |
|  | C | 0.65 | 0.57 | 0.56 | - | 0.80 | 0.49 | -0.22 | 0.44 | 0.58 | 0.42 |
|  | 28-Item | 0.88 | 0.82 | 0.80 | 0.84 | - | 0.47 | 0.01 | 0.54 | 0.44 | 0.56 |
|  | S | 0.50 | 0.42 | 0.39 | 0.59 | 0.61 | - | 0.00 | 0.56 | 0.58 | 0.78 |
|  | CRF | 0.18 | 0.31 | 0.19 | 0.29 | 0.27 | 0.41 | - | 0.14 | -0.11 | 0.22 |
| MTB | F | 0.43 | 0.38 | 0.59 | 0.41 | 0.55 | 0.50 | 0.26 | - | 0.45 | 0.86 |
|  | C | 0.40 | 0.35 | 0.33 | 0.53 | 0.49 | 0.62 | 0.32 | 0.34 | - | 0.68 |
|  | 28-Item | 0.47 | 0.44 | 0.51 | 0.51 | 0.58 | 0.82 | 0.56 | 0.84 | 0.70 | - |

*Abbreviation:* N = sample size, S = Muscular strength, CRF = cardiorespiratory fitness, F = flexibility, C = Gross motor coordination, MTB = motor test battery, r = correlation coefficient, * = p < 0.05.

Light grey cells = males (N = 228); white cells = females (N = 228)

*Norms:* Percentile and stanine norms exist based on data of 458 persons (229 men, 229 women) aged 30 to 65 years. Norm tables are available upon request from the corresponding author.
